# Exploring Beliefs, Concerns, Prenatal Care Advice, and Sources of Information About Gestational Weight Gain Among Immigrant Central American Pregnant Women in the United States

**DOI:** 10.3390/ijerph21121672

**Published:** 2024-12-14

**Authors:** Virginia A. Moreno, Doris Lucero, Nachalie Rodriguez-Cruz, Qun Le, Mary L. Greaney, Ana Cristina Lindsay

**Affiliations:** 1 Department of Exercise and Health Sciences, Robert J and Donna Manning College of Nursing and Health Sciences, University of Massachusetts, Boston, MA 02125, USA; virginia.arango001@umb.edu (V.A.M.); n.rodriguezcruz001@umb.edu (N.R.-C.); 2 Department of Biology, College of Sciences and Mathematics, University of Massachusetts, Boston, MA 02125, USA; doris.lucero001@umb.edu; 3 Department of Public Health, Zuckerberg College of Health Sciences, University of Massachusetts, Lowell, MA 01854, USA; qun_le@uml.edu; 4 Department of Public Health, College of Health Sciences, University of Rhode Island, Kingston, RI 02881, USA; mgreaney@uri.edu; 5 Department of Urban Public Health, Robert J and Donna Manning College of Nursing and Health Sciences, University of Massachusetts, Boston, MA 02125, USA

**Keywords:** pregnancy, gestational weight gain, beliefs, information, Central American, United States

## Abstract

Gestational weight gain (GWG) is critical for maternal and neonatal health, but excessive GWG can lead to complications such as gestational diabetes, hypertension, and increased obesity risk later in life. Minoritized and immigrant women often face higher risks of excessive GWG. This cross-sectional study assessed Central American women’s beliefs and concerns about GWG, the receipt of advice from healthcare providers, and sources of information for healthy weight management during pregnancy. A cross-sectional survey was conducted with 93 pregnant women from El Salvador (31.2%), Guatemala (46.2%), and Honduras (22.6%). Most participants were married (91.4%), and 91.2% had household incomes below $40,000. Self-reported pre-pregnancy weight status varied significantly (*p* = 0.03), with more Guatemalans self-reporting as overweight (34.9%) compared to Salvadorans (10.3%) and Hondurans (19.1%). Beliefs about GWG varied significantly; 72.1% of Guatemalan women accepted “eating for two”, while only 31.0% of Salvadorans did (*p* = 0.002). More Honduran women (90.5%) received weight gain recommendations from healthcare providers than Salvadorans (62.1%) and Guatemalans (60.5%) (*p* = 0.04). The Internet and family were common information sources on weight management, highlighting the need for culturally tailored health education. This study underscores critical differences in beliefs and access to prenatal care among pregnant Central American immigrant women, emphasizing the importance of culturally competent health education to support healthy pregnancy outcomes.

## 1. Introduction

Gestational weight gain (GWG) plays a crucial role in the health of both the mother and the newborn [1,2,3]. However, excessive GWG increases the risk of maternal complications, including gestational diabetes, pregnancy-induced hypertension (such as preeclampsia), labor and delivery issues (like the need for a cesarean section), postpartum weight retention, and long-term health risks such as heart disease and diabetes [4,5]. For the newborn, excessive GWG raises the likelihood of childhood obesity, metabolic disorders, and adverse birth outcomes [4,5,6]. Thus, monitoring and managing GWG is essential for ensuring healthy pregnancy outcomes and promoting the long-term health of both mother and child.

According to the Centers for Disease Control and Prevention (CDC), approximately 47% of pregnant women in the United States (U.S.) gain more weight during pregnancy than recommended by the 2009 Institute of Medicine (IOM) guidelines for GWG, which are based on pre-pregnancy body mass index (BMI) categories [7,8,9,10]. This is concerning, especially among minoritized and immigrant women, with research indicating that over 60% of U.S.-born Hispanic women and 52% of non-Hispanic Black women exceed the recommended GWG guidelines [11,12,13]. Contributing factors include limited access to healthcare, cultural beliefs about nutrition and pregnancy, and socioeconomic challenges [14,15,16,17]. Additionally, immigrant women often rely on informal sources of information regarding weight gain during pregnancy, which may exacerbate the risk of excessive GWG [18,19,20,21].

Healthcare providers have a critical role in addressing GWG, as their guidance on GWG, diet, physical activity (PA), and exercise can significantly influence the behaviors and beliefs of pregnant women [21,22,23,24]. For ethnic minoritized and immigrant populations, such as Central Americans, culturally sensitive counseling from healthcare providers is essential for dispelling myths, addressing incorrect beliefs, and promoting healthy practices that are tailored to their unique cultural contexts [22,23,24,25]. As these populations navigate living in a new country and barriers to care, they may encounter conflicting health information from their home culture and the host society, underscoring the importance of providing evidence-based, culturally relevant guidance [19,21,24]. When healthcare providers offer clear, evidence-based advice, they empower women to make informed health decisions, ultimately enhancing maternal and neonatal outcomes [21,26]. Improving access to quality prenatal care and strengthening provider–patient communication are critical strategies for reducing the risks associated with excessive GWG in these vulnerable populations [26,27].

Despite their notable presence in the U.S., the beliefs, concerns, and information sources about GWG among pregnant Central American immigrant women remain underexplored [28]. Understanding these beliefs, the sources of information, and how healthcare provider advice influences GWG-related behaviors is crucial for developing effective, tailored health interventions for this population. This study aimed to assess Central American women’s beliefs and concerns about GWG, examine the receipt of healthcare provider guidance, and identify the information sources they use to learn about healthy weight management during pregnancy. 

## 2. Materials and Methods

### 2.1. Sample and Setting

A cross-sectional design was employed with a convenience sample of Central American pregnant women from El Salvador, Guatemala, and Honduras residing in Massachusetts (MA), U.S. Trained researchers conducted a telephone interview using a paper survey from May to September 2021, data collection spanned both the spring and summer months in the U.S. 

Inclusion criteria included (a) being a native-born Salvadoran, Guatemalan, or Honduran; (b) being 18 years of age or older; (c) being pregnant with a single birth; (d) being at least at 14 weeks of gestation; (e) residing in MA; (f) speaking Spanish or English; and (g) providing informed consent.

### 2.2. Recruitment

Participants were recruited through community organizations, social media, local events, and network sampling, where enrolled participants referred others [28,29,30]. Recruitment efforts involved community outreach, social media platforms, and the engagement of research staff who were members of the Central American communities participating in the study [28,30]. These staff members leveraged their personal and community contacts to facilitate recruitment [28,30]. All interested individuals were assessed for eligibility either in person or by telephone by study staff. Interested persons contacted study staff using the phone number listed on the flyer or spoke with them at local community and church events.

### 2.3. Data Collection and Survey Measures

After confirming eligibility, but before enrollment, eligible participants were read the informed consent form in their preferred language (Spanish or English) by a trained bilingual interviewer. Upon providing verbal consent, participants completed a brief survey in their preferred language, administered by the interviewer over the telephone. Data were collected by bilingual interviewers trained in survey content, interviewing techniques, and cultural competency.

The survey, informed by the literature [18,20,23], included 72 items adapted from previous studies [31,32,33,34]. The survey assessed seven domains: (1) beliefs and concerns about the safety of GWG and weight management strategies (seven questions); (2) knowledge and perceptions of postpartum weight loss (four questions); (3) information received from their healthcare provider as that is how you talk about it later during prenatal care (four questions); (4) sources used to seek information during pregnancy (nine questions); (5) self-perception of current GWG (two questions); (6) oral health beliefs (16 questions); and (7) COVID-19 concerns, vaccine uptake, and hesitancy (30 questions). The present study specifically focuses on participants’ responses to questions assessing beliefs, health concerns, prenatal care advice, and sources of information about GWG. The survey was translated into Spanish and pilot tested with three pregnant women who are not included in the final sample. 

Additionally, the survey collected demographic and socioeconomic data, including age, gender, marital status, education, income, employment, health insurance status, country of birth, length of U.S. residence, enrollment in the Special Supplemental Nutrition Program for Women, Infants, and Children (WIC), parity, and acculturation level assessed using the Short Acculturation Scale for Hispanics (SASH) [18]. This 12-item scale measures language use, media consumption, and ethnic social relations, demonstrating high reliability (Cronbach’s alpha: 0.92–0.72) [35,36]. Acculturation scores were averaged (range 1–5), with scores of 2.99 or above indicating higher acculturation [35].

Data collection occurred between May and September, spanning the spring and summer months. Participants received a $25 gift card as compensation for their participation.

### 2.4. Data Analysis

Descriptive statistics (means, frequencies, percentages) summarized demographic and socioeconomic characteristics (age, gender, length of U.S. residence, type of health insurance, acculturation, etc.), health-related characteristics, beliefs, healthcare providers’ advice, and prenatal care information sources. χ^2^ and Fisher’s exact tests were used as appropriate to determine if there were differences in beliefs and concerns about GWG, healthcare providers’ advice, sources used to seek information during pregnancy, self-reported pre-pregnancy weight status, sociodemographic variables, acculturation, type of health insurance by country of origin (El Salvador, Guatemala, or Honduras). All analyses were conducted with SAS 9.4, using a *p* < 0.05 to determine significance [37].

## 3. Results

### 3.1. Socio-Demographic and Health-Related Characteristics

As seen in Table 1, all participants (*n* = 93) were foreign-born, with 32.1% (*n* = 29) being Salvadoran, 46.2% Guatemalan (*n* = 43), and 22.6% Honduran (*n* = 21). Most participants (91.4%) were married or cohabiting, and 91.2% reported household incomes below US $40,000, with no significant differences between these variables by country of origin. Educational attainment was also similar across the groups, with 83.9% of women having more than a high school education. The analysis determined a significant difference in age by country of origin, with Guatemalans being the youngest (mean age 29.4 years) compared to Salvadorans and Hondurans (both with a mean age of 32.8 years, *p* = 0.01).

The mean years of residence in the U.S. were comparable across groups, with no significant differences observed (*p* = 0.19). More than half of the participants (59.1%) had lived in the U.S. for over 10 years. Most participants (76.3%) scored below 2.99 on the SASH, indicating low acculturation levels, and there were no significant differences between groups (*p* = 0.32). Additionally, most participants (78.5%) had government-sponsored health insurance, and 41.9% were enrolled in the WIC program, and there were no significant differences by group (*p* = 0.19, *p* = 0.29, respectively).

Most participants (73.1%) reported that this was their first pregnancy, and there were no significant differences by country of origin (*p* = 0.66). The mean number of gestation weeks was similar across groups, with Salvadorans averaging 23.7 (SD = 8.2) weeks, Guatemalans 23.0 (SD = 5.4) weeks, and Hondurans 23.5 (SD = 6.50 weeks), indicating no significant differences (*p* = 0.90). 

Lastly, perceived pre-pregnancy weight status also differed significantly by group (*p* = 0.03), with more Guatemalans self-reporting being overweight (34.9%) compared to Salvadorans (10.3%) and Hondurans (19.1%).

### 3.2. Beliefs, Concerns, and Knowledge About GWG

As shown in Table 2, a little more than half of the women (53.8%) believed that is “fine to eat as much as you want when you are pregnant, you are ‘eating for two’”. There was a significant difference by group with a higher proportion of Guatemalan women (72.1%) believing that that is acceptable during pregnancy, compared to 47.6% of Honduran women and 31.0% of Salvadoran women (*p* = 0.002). 

In addition, nearly all Salvadoran women (96.6%) believed that exercise is safe during pregnancy, compared to 81.0% of Honduran women and 51.2% of Guatemalan women, and this difference was statistically significant (*p* < 0.0001). A higher percentage of Honduran women (33.3%) believed that dieting is safe during pregnancy compared to Guatemalan women (25.6%) and Salvadoran women (17.2%), though the difference was not statistically significant (*p* = 0.42).

Most women across the three groups expressed concern about gaining too much weight during pregnancy, with 86.2% of Salvadoran, 76.2% of Honduran, and 69.8% of Guatemalan women sharing this concern. However, these differences were not significant (*p* = 0.32). A high percentage of women acknowledged the medical risks for themselves (87.1%) and their baby (86.0%) if they gained too much weight during pregnancy, and there were no significant differences between groups (*p* = 0.57 and *p* = 0.80, respectively). Nearly all Guatemalan women (97.7%) were interested in learning more about losing their “baby weight” after delivery, compared to 82.8% of Salvadoran women and 76.2% of Honduran women (*p* = 0.01), indicating a significant difference in interest levels by group.

### 3.3. Prenatal Care Advice Received from Health Care Professionals 

As shown in Table 3, most women (67.7%) reported that their healthcare provider (midwife or doctor) discussed how much weight they should try to gain during pregnancy. Many (74.2%) women indicated that their healthcare provider advised them on what to eat during pregnancy. Additionally, most (64.5%) of the women reported receiving advice on exercise during pregnancy from their healthcare providers. 

Several key differences were identified in prenatal care advice received by Salvadoran, Guatemalan, and Honduran women (Table 3). More Honduran women (90.5%) than Salvadoran (62.1%) and Guatemalan (60.5%) women reported that their healthcare provider recommended how much weight they should gain during pregnancy, and this difference was significant (*p* = 0.04). Although not statistically significant, 90.5% of Honduran women received advice on gaining the right weight during pregnancy, compared to 69.0% of Salvadorans and 62.8% of Guatemalans (*p* = 0.06). There was no significant difference between groups regarding receiving exercise advice during pregnancy (76.2% of Hondurans, 72.4% of Salvadorans, and 53.5% of Guatemalans (*p* = 0.12). A significantly higher proportion of Honduran women (95.2%) received advice on what to eat during pregnancy, compared to 72.4% of Salvadorans and 65.1% of Guatemalans (*p* = 0.02).

### 3.4. Sources of Information Related to Weight Gain, Diet and Nutrition, and PA and Exercise During Pregnancy

Table 4 presents information on the information sources participants used for information related to weight gain, diet and nutrition, and PA/exercise during their pregnancy. The results identified a common approach to obtaining health information during pregnancy across the three groups (Salvadoran, Guatemalan, and Honduran), with no significant differences in sources of information. 

Overall, results showed that participants relied on interpersonal sources of information, with most women turning to family members and friends for information about weight gain, PA/exercise, and nutrition during pregnancy. About two-thirds of women (73.1%) turned to family members and friends for information about weight gain during pregnancy, and there were no significant differences between groups (*p* = 0.63). Similarly, most women sought advice from family members (67.7%) and friends (74.2%) about diet, with no significant difference between groups (*p* = 0.87 and *p* = 0.95, respectively). Talking to family (61.3%) and friends (59.1%) about PA/exercise during pregnancy was also common among women without significant differences between groups (*p* = 0.53 and *p* = 0.39, respectively). 

While there was a substantial reliance on interpersonal sources, a notable proportion of participants also utilized the Internet to seek information on various pregnancy-related topics. Over half (57%) of the women searched the Internet for information on weight gain during pregnancy, although there were no differences by group (*p* = 0.77). Many women also used the Internet to search for information about PA/exercise (64.5%) and diet and nutrition information (69.9%), with Honduran women showing slightly higher usage at 81.0%, although these differences were not significant (*p* = 0.20 and *p* = 0.49, respectively).

## 4. Discussion

The purpose of this study was to assess pregnant Central American women’s beliefs and concerns about GWG, examine the receipt of healthcare provider guidance, and identify the information sources they use to learn about healthy weight management during pregnancy. Overall, the study found that most women were concerned about gaining too much weight during pregnancy, and nearly all acknowledged the health risks associated with excessive weight gain for themselves and their babies. While about half of the participants believed it was acceptable to “eat for two” during pregnancy, the majority expressed interest in learning about postpartum weight loss. Most participants reported receiving prenatal care advice on weight gain, diet and nutrition, and PA/exercise primarily from healthcare providers (midwife or doctors), although women also sought information from interpersonal sources (e.g., friends and family) and the Internet.

### 4.1. Demographic and Socio-Cultural Characteristics 

The sample’s demographic characteristics showed variations in maternal age, with Guatemalan women being significantly younger than their Salvadoran and Honduran counterparts. This age difference may affect pregnancy-related health behaviors and needs, as younger women might require more targeted health education and support [38,39]. Despite age differences, the groups shared similarities in socio-economic factors, such as household income and educational attainment, suggesting that economic challenges are common across Central American immigrant populations in the U.S. Previous studies have demonstrated the critical role of social determinants of health, such as income and education, in influencing GWG, highlighting the importance of addressing these factors to improve maternal and child health outcomes [39,40]. 

Moreover, most women in this study had low acculturation levels, highlighting the need for culturally appropriate health communication strategies that resonate with their cultural contexts and experiences. Lower levels of acculturation have been associated with variations in GWG, as acculturated women may adopt different dietary patterns and health behaviors during pregnancy, which can impact weight gain [40,41]. Given the low levels of acculturation across the sample, culturally appropriate health communication strategies are essential. Healthcare providers should develop education materials and programs that resonate with Central American immigrant populations’ cultural values, beliefs, and experiences, particularly focusing on maternal and child health behaviors [40,41,42,43]. Addressing social determinants like income and education should also be integrated into health interventions to improve overall outcomes [40,44,45].

### 4.2. Beliefs and Concerns About GWG

More than two-thirds (76.3%) of the women participating in this study expressed concern about gaining too much weight during pregnancy. Similarly, almost two-thirds (72%) felt that PA/exercise is safe during pregnancy. The high level of concern regarding excessive weight gain suggests a need for targeted educational interventions that address healthy weight management during pregnancy. These programs could provide women with evidence-based diet, nutrition, and PA/exercise information, helping alleviate fears and promote healthy practices. Additionally, given that a large proportion of participants view exercise as safe during pregnancy, there is an opportunity to promote PA as a beneficial component of prenatal care. These findings are important and suggest that healthcare providers can encourage, and support safe exercise routines tailored to the needs and abilities of pregnant women, potentially improving both maternal and fetal health outcomes [46,47,48,49].

The study also identified significant differences in beliefs about GWG, particularly regarding the perception that it is acceptable to “eat for two” during pregnancy. A greater percentage of Guatemalan women held this belief, which could be associated with cultural norms or misconceptions about pregnancy nutrition [40,41,42,50]. These findings suggest a critical opportunity for healthcare providers to deliver culturally sensitive counseling that dispels myths and promotes healthy weight management during pregnancy [32,43,51,52]. In contrast, Salvadoran women were more likely to believe that exercise is safe during pregnancy, which is encouraging given the known benefits of exercise for maternal and fetal health. However, the lower belief in exercise safety among Guatemalan women indicates a potential area for intervention, as addressing these concerns could enhance maternal health outcomes [25,44,46]. Prior studies conducted among Latina women show that cultural beliefs often influence their perceptions of appropriate weight gain during pregnancy, with many believing that gaining excess weight reflects a healthy pregnancy and better fetal outcomes [50,51,52,53,54,55,56].

### 4.3. Health Care Professional Advice and Information Sources

Most participants (67.7%) reported that their healthcare provider recommended how much weight they should try to gain during pregnancy. This suggests that healthcare providers play a key role in offering guidance on weight management during pregnancy. In addition, about two-thirds (74.2%) of the women indicated that their healthcare provider gave them advice on what to eat during pregnancy, highlighting the importance of healthcare providers in providing dietary guidance. These findings are important, as previous studies suggest the importance of healthcare providers’ advice regarding GWG and diet and nutrition to reduce the risk of excessive GWG among pregnant women [22,26], including Latinas [43,46]. 

However, a notable study finding is the significantly higher percentage of Honduran women who received prenatal care advice from healthcare professionals compared to Salvadoran and Guatemalan women. It is possible that Honduran women have better access to prenatal care services or more consistent communication with their healthcare providers regarding GWG, diet and nutrition, and PA/exercise. It is important to point out that although not statistically significant, there were differences in enrollment in the WIC program, with more Honduran women (52.4%) being enrolled compared to Guatemalan (44.2%) and Salvadoran (31%) women. WIC enrollment may serve as an indicator of greater access to healthcare services, as the program provides nutritional counseling and support, which could contribute to the observed differences in prenatal care advice. In contrast, the lower percentage of Salvadoran and Guatemalan women receiving this advice is indicative of a gap in prenatal care that could negatively affect pregnancy outcomes. This underscores the importance of ensuring equitable access to high-quality prenatal care and reinforcing healthcare providers’ role in offering tailored guidance to all pregnant Central American women [46,47,51,52,56,57]. Previous studies underscore the critical role of healthcare providers in advising patients on appropriate GWG, as this guidance is essential for promoting maternal and fetal health and reducing the risk of complications during pregnancy [56,57].

The study also revealed that pregnant Central American immigrant women in this study frequently sought information about weight gain, diet, and PA/exercise from family members, friends, and the Internet. This reliance on informal sources of information was prevalent and consistent across the groups, suggesting that while healthcare providers play a critical role, these informal networks are also influential in shaping women’s pregnancy-related beliefs and behaviors. This finding is supported by previous studies that found that pregnant women rely on a combination of healthcare advice and informal sources, such as family and peers, to guide their decisions regarding health and wellness [18,19,20]. Public health interventions that leverage these trusted social networks could effectively disseminate accurate health information. Additionally, the widespread use of the Internet for health-related information highlights the potential for digital health interventions targeting Central American women, particularly through culturally and linguistically appropriate online resources. Assessing and increasing the health literacy of these women is also critical, equipping them with the skills to navigate and assess online health information effectively. Furthermore, training healthcare providers on how to address GWG in culturally sensitive ways will enhance their ability to offer personalized, relevant guidance. Ensuring the availability and accessibility of culturally and linguistically sensitive educational materials in healthcare offices is also essential to support these women during pregnancy and postpartum [58,59,60,61,62,63].

### 4.4. Weight Concerns and Interest in Postpartum Weight Loss

Most women (76.3%) expressed concern about gaining too much weight during pregnancy, with Salvadoran women showing the highest level of concern. This finding is consistent with previous studies showing that women from immigrant backgrounds are often aware of the potential health risks of excessive GWG [11,21,31,42]. Interestingly, Guatemalan women expressed a significantly higher interest in learning about postpartum weight loss than Salvadoran and Honduran women, suggesting that educational programs focused on postpartum health and weight management could be particularly beneficial for this group [41,60,61,62,63]. 

### 4.5. Limitations and Strengths of the Study

The findings of this study should be interpreted with several limitations in mind. First, the cross-sectional design, lack of a priori sample size calculations, and small sample size limit the robustness of the results [64]. Second, pre-pregnancy weight status was based on self-reported information, which may underestimate true pre-pregnancy status and bias findings [65]. Additionally, although data were collected during pregnancy, the outcome variables were based on self-reported information, making them vulnerable to recall bias [63]. The study did not collect data on diet, dietary patterns, PA/exercise, prenatal care routines, or the clinical settings where prenatal care was received. This information could have provided additional valuable insights and should be collected in future research. Finally, the unique characteristics of the study setting and sample further constrain the generalizability of the results [64]. 

Despite these limitations, the study has notable strengths, including its focus on an understudied ethnic sample of Salvadoran, Guatemalan, and Honduran immigrant women, and the use of an acculturation measure (SASH) [35,36]. These factors are crucial for designing interventions that meet the sociocultural needs of ethnic minority immigrant populations. Additionally, while the study’s focus is not entirely novel, it is one of the first to specifically examine pregnant Central American immigrant women living in the U.S. Future research with a larger sample size is needed to explore the associations between sociocultural variables, pre-pregnancy weight status, and healthcare providers’ advice on GWG, diet, PA, and exercise.

## 5. Public Health Implications and Recommendations

Based on study findings, several public health implications and recommendations can be derived to enhance maternal health outcomes for pregnant Central American immigrant women.

### 5.1. Need for Culturally Tailored Health Education and Counseling

The significant differences in beliefs about GWG among Guatemalan, Honduran, and Salvadoran women underscore the need for culturally tailored interventions. Healthcare providers should prioritize:

- Dispelling myths such as the belief that it is acceptable to “eat for two” during pregnancy, particularly among Guatemalan women.
- Promoting safe exercise practices, with a focus on addressing concerns in groups like Guatemalan women, who showed lower belief in the safety of exercise during pregnancy.
- Educating on nutrition and appropriate weight gain to ensure that women are equipped with accurate information, especially given the high reliance on family members and the Internet for health advice.

### 5.2. Leveraging Informal Networks for Health Messaging

Given the frequent use of informal sources (family, friends, and the Internet) by pregnant women across all three groups, public health interventions should:

- Engage family members in health education efforts, as they are influential in shaping beliefs about weight gain, PA/exercise, and diet during pregnancy.
- Develop digital health campaigns that are culturally and linguistically appropriate, providing accurate and accessible information on GWG, nutrition, and postpartum weight loss through trusted online platforms.
- Teach women skills to identify trusted sites for information, empowering them to differentiate between reliable and unreliable online health sources, ensuring they access accurate and evidence-based content.

### 5.3. Improving Access to Consistent Prenatal Care Advice

The disparity in the receipt of prenatal care advice, particularly the higher percentage of Honduran women receiving advice compared to Salvadoran and Guatemalan women, highlights the need for:

- Ensuring equitable access to high-quality prenatal care across all Central American immigrant groups. This includes consistent guidance on GWG, diet, and PA, particularly for groups that may have less interaction with healthcare providers.
- Strengthening healthcare providers’ role in offering personalized and culturally sensitive counseling, especially for Guatemalan women, who were less likely to receive advice on weight management and PA/exercise during pregnancy.
- Leveraging the Women, Infants, and Children (WIC) program as an additional source of prenatal care advice, providing guidance on nutrition, weight gain, and healthy behaviors during pregnancy to ensure that women from all backgrounds receive accurate and consistent information.

### 5.4. Postpartum Health and Weight Management Programs

The high interest in postpartum weight loss, particularly among Guatemalan women, suggests the need for:

- Addressing postpartum health disparities by ensuring that all pregnant Central American women, regardless of country of origin, have access to resources and information on how to maintain a healthy weight after pregnancy.
- Developing postpartum health programs focused on weight management and healthy lifestyle behaviors. These programs could be integrated into existing maternal health services, providing continued support to women after delivery.
- Designing postpartum programs that are culturally appealing and effectively marketed to this population, ensuring that the content resonates with their specific needs and motivations, making it more likely that they will engage with and benefit from the services.

### 5.5. Addressing Social Determinants of Health

The study highlights common socio-economic challenges faced by pregnant Central American immigrant women, such as low income and educational attainment. Public health initiatives should:

- Incorporate social support services into prenatal care programs to address economic barriers that may limit access to nutritious food, PA/exercise options, and healthcare services.
- Target low acculturation levels with interventions that account for cultural and language differences, helping to bridge gaps in health literacy and support healthier pregnancy outcomes.
- Ensure that health care organizations are health literate, meaning that patients are empowered to find, navigate, and use the information to promote health.

### 5.6. Expanding Research and Future Interventions

The study’s findings reveal gaps in understanding how sociocultural variables, such as acculturation and pre-pregnancy weight status, influence health behaviors and outcomes. Future public health research should:

- Investigate larger, more diverse samples to explore these relationships further, which can help refine interventions that promote healthy GWG and improve maternal health outcomes in Central American immigrant populations.
- Examine long-term health outcomes, including postpartum weight retention and child health, to develop comprehensive maternal health programs that address both pregnancy and postpartum needs.

By addressing these gaps and building on the study’s findings, public health efforts can better support the diverse and complex needs of Central American immigrant women during pregnancy and beyond.

## 6. Conclusions

In conclusion, this study highlights critical differences in beliefs, concerns, receipt of prenatal care advice, and sources of information used by pregnant Central American immigrant women, emphasizing the need for culturally competent health education and interventions. By addressing these disparities and leveraging informal networks and digital platforms, public health programs can better support Central American women in achieving healthy pregnancy outcomes and promoting long-term maternal health.

## Figures and Tables

**Table 1:** Demographic, socioeconomic, and health-related characteristics of the study sample (*n* = 93).

Variables	Total *n* = 93 (%)	Salvadoran *n* = 29 (%)	Guatemalan *n* = 43 (%)	Honduran *n* = 21 (%)	*p*-Value
**Number Weeks Gestation, Mean, (SD)**	23.3 (6.6)	23.7 (8.2)	23.0 (5.4)	23.5 (6.5)	0.90
**Age, mean (SD)**	31.2 (5.4)	32.8 (3.5)	29.4 (5.6)	32.8 (6.1)	0.01 **
≤25 years	12 (12.9)	2 (6.9)	7 (16.3)	3 (14.3)	0.21
<25–<35 years	56 (60.2)	17 (58.6)	29 (67.4)	10 (47.6)	
≥35 years	25 (26.9)	10 (34.5)	7 (16.3)	8 (38.1)	
**Marital status**					
Married/Living together	85 (91.4)	26 (89.7)	39 (90.7)	20 (95.2)	0.28
Separated/Divorced	3 (3.2)	0 (0.0)	3 (7.0)	0 (0.0)	
Single	5 (5.4)	3 (10.3)	1 (2.3)	1 (4.8)	
**Household income**					
<US $40,000/year	62 (91.2)	21 (87.5)	28 (96.6)	13 (86.7)	0.35
≥US $40,000/year	6 (8.8)	3 (12.5)	1 (3.5)	2 (13.3)	
**Educational attainment**					
<High school diploma	15 (16.1)	4 (13.8)	8 (18.6)	3 (14.3)	0.93
≥High school graduate	78 (83.9)	25 (86.2)	35 (81.4)	18 (85.7)	
**Foreign-born**					
Yes	93 (100.0)	29 (100.0)	43 (100.0)	21 (100.0)	-
**Years of residence in the U.S., mean (SD)**	10.7 (6.1)	12.2 (6.3)	10.5 (5.6)	9.1 (6.4)	0.19
<10 years	38 (40.9)	9 (31.0)	17 (39.5)	12 (57.1)	0.17
≥10 years	55 (59.1)	20 (69.0)	26 (60.5)	9 (42.9)	
**SASH score**					
<2.99	71 (76.3)	25 (86.2)	30 (69.8)	16 (76.2)	0.32
≥2.99	22 (23.7)	4 (13.8)	13 (30.2)	5 (23.8)	
**Health insurance**					
Government-sponsored	73 (78.5)	26 (89.7)	32 (74.4)	15 (71.4)	0.19
Private	20 (21.5)	3 (10.3)	11 (25.6)	6 (28.6)	
Uninsured	0 (0.0)	0 (0.0)	0 (0.0)	0 (0.0)	
**Enrollment in the WIC program**					
Yes	39 (41.9)	9 (31.0)	19 (44.2)	11 (52.4)	0.29
No	54 (58.1)	20 (69.0)	24 (55.8)	10 (47.6)	
**First pregnancy**					
Yes	68 (73.1)	21 (72.4)	30 (69.8)	17 (81.0)	0.66
No	25 (26.9)	8 (27.6)	13 (30.2)	4 (19.0)	
**Pre-pregnancy self-reported weight status**					
Underweight	9 (9.7)	1 (3.5)	6 (14.0)	2 (9.5)	0.03 *
Healthy/Normal Weight	61 (65.6)	25 (86.2)	22 (51.2)	14 (66.7)	
Overweight	22 (23.7)	3 (10.3)	15 (34.9)	4 (19.1)	
Obese	1 (1.08)	0 (0.0)	0 (0.0)	1 (4.8)	

Note: SD = standard deviation; SASH = Short Acculturation Scale for Hispanics; *p* < 0.05 *, *p* < 0.01 **.

**Table 2:** Beliefs and knowledge related to gestational weight gain (GWG) and postpartum weight loss.

Beliefs About the Safety of GWG and Weight Management Strategies					
Variables	Total *n* = 93 (%)	Salvadoran *n* = 29 (%)	Guatemalan *n* = 43 (%)	Honduran *n* = 21 (%)	*p*-Value
It is fine to eat as much as you want when you are pregnant, you are “eating for two”	50 (53.8)	9 (31.0)	31 (72.1)	10 (47.6)	0.002 **
Exercise is safe during pregnancy	67 (72.0)	28 (96.6)	22 (51.2)	17 (81.0)	<0.0001 ***
Dieting is safe during pregnancy	23 (24.7)	5 (17.2)	11 (25.6)	7 (33.3)	0.42
Diet pills are safe during pregnancy	6 (6.5)	0 (0.0)	5 (11.6)	1 (4.8)	0.10
It is safe to lose weight in pregnancy	14 (15.1)	4 (13.8)	8 (18.6)	2 (9.5)	0.75
I am worried about gaining too much weight during pregnancy	71 (76.3)	25 (86.2)	30 (69.8)	16 (76.2)	0.32
I am having trouble gaining weight during pregnancy	30 (32.3)	7 (24.1)	14 (32.6)	9 (42.9)	0.38
**Knowledge of Maternal and Fetal Risks of Excessive GWG and Postpartum Weight Loss**					
There are medical risks for me if I gain too much weight during pregnancy	81 (87.1)	25 (86.2)	39 (90.7)	17 (81.0)	0.57
There are medical risks for my baby if I gain too much weight during pregnancy	80 (86.0)	24 (82.8)	38 (88.4)	18 (85.7)	0.80
Breastfeeding will help me lose my “baby weight”	68 (73.1)	22 (75.9)	30 (69.8)	16 (76.2)	0.80
I would be interested in learning more about how to lose my “baby weight” after I deliver	82 (88.2)	24 (82.8)	42 (97.7)	16 (76.2)	0.01 *

*p* < 0.05 *, *p* < 0.01 **, *p* < 0.001 ***.

**Table 3:** Information received from healthcare professionals during prenatal care.

Information Topics	Total *n* = 93 (%)	Salvadoran *n* = 29 (%)	Guatemalan *n* = 43 (%)	Honduran *n* = 21 (%)	*p*-Value
My midwife or doctor recommended how much weight I should try to gain during pregnancy	63 (67.7)	18 (62.1)	26 (60.5)	19 (90.5)	0.04 *
My midwife or doctor gave me advice on how to gain the right amount of weight during pregnancy	66 (71.0)	20 (69.0)	27 (62.8)	19 (90.5)	0.06
My midwife or doctor gave me advice on exercise during pregnancy	60 (64.5)	21 (72.4)	23 (53.5)	16 (76.2)	0.12
My midwife or doctor gave me advice on what to eat during pregnancy	69 (74.2)	21 (72.4)	28 (65.1)	20 (95.2)	0.02 *

Note: *p* < 0.05 *.

**Table 4:** Sources of information sought by women related weight gain, diet and nutrition, and PA/exercise during pregnancy.

Information Sources	Total *n* = 93 (%)	Salvadoran *n* = 29 (%)	Guatemalan *n* = 43 (%)	Honduran *n* = 21 (%)	*p*-Value
I use(d) the Internet to search for information about weight gain during pregnancy	53 (57.0)	18 (62.1)	23 (53.5)	12 (57.1)	0.77
I use(d) the Internet to search for information about physical activity and exercise during pregnancy	60 (64.5)	18 (62.1)	25 (58.1)	17 (81.0)	0.20
I use(d) the Internet to search for information about diet and nutrition during pregnancy	65 (69.9)	19 (65.5)	29 (67.4)	17 (81.0)	0.49
I talk(ed) to family members to get information about weight gain during pregnancy	68 (73.1)	20 (69.0)	31 (72.1)	17 (81.0)	0.63
I talk(ed) to friends to get information about weight gain during pregnancy.	68 (73.1)	22 (75.9)	30 (69.8)	16 (76.2)	0.80
I talk(ed) to family members to get information about physical activity and exercise during pregnancy	57 (61.3)	20 (69.0)	24 (55.8)	13 (61.9)	0.53
I talk(ed) to friends to get information about physical activity and exercise during pregnancy	55 (59.1)	17 (58.6)	23 (53.5)	15 (71.4)	0.39
I talk(ed) to family members to get information about diet and nutrition during pregnancy	63 (67.7)	20 (69.0)	28 (65.1)	15 (71.4)	0.87
I talk(ed) to friends to get information about diet and nutrition during pregnancy.	69 (74.2)	21 (72.4)	32 (74.4)	16 (76.2)	0.95

## Data Availability

Due to privacy or ethical restrictions, the data that support the findings of this analysis are not publicly available; however, they are available upon request from the corresponding author.
